# Regional variation in care at the end of life: discontinuation of dialysis

**DOI:** 10.1186/1471-2318-13-39

**Published:** 2013-05-01

**Authors:** Charles E Gessert, Irina V Haller, Brian P Johnson

**Affiliations:** 1 Division of Research, Essentia Institute of Rural Health, 502 East 2nd Street, Duluth, MN, USA

**Keywords:** Regional variation, Dialysis, End-stage renal disease, End-of-life care

## Abstract

**Background:**

Regional variation in the intensity of end-of-life care contributes significantly to the overall cost of health care. The interpretation of patterns of regional variation hinges, in part, on appropriate adjustment for regional variation in demographic variables such as age, race, sex, and rural vs. urban residence. This study examined regional variation in discontinuation of dialysis prior to death in the US, after adjustment for key demographic variables.

**Methods:**

In this retrospective cohort study of the 2009 United States Renal Data System (USRDS) database we examined discontinuation of dialysis prior to death among deceased adult patients with end-stage renal disease (ESRD) from the 50 states and the District of Columbia. The discontinuation of dialysis prior to death was ascertained from the Centers for Medicare & Medicaid Services form 2746 (ESRD Death Notification form). We used logistic regression to estimate the log-odds of discontinuation of dialysis with ESRD network as independent variable adjusted for urban–rural status, demographic and treatment variables.

**Results:**

The study cohort included 715,605 deceased ESRD patients; for 176,021 of whom (24.6%) dialysis was discontinued prior to death. Dialysis was discontinued at higher rates for women than for men (26.3% vs. 23.0%, p < 0.001) and for whites than for blacks (29.5% vs. 14.7%, p < 0.001). Significant regional variation in dialysis discontinuation prior to death was noted after adjustment for age, race and rural–urban status: rates of discontinuation in the Upper Midwest and Mountain regions were more than double the rates in Southern and Coastal regions. This pattern parallels the regional pattern of end-of-life health service utilization documented in the Dartmouth Atlas and other studies.

**Conclusions:**

Discontinuation of dialysis prior to death was common in the US between 1995 and 2009. The deaths of nearly one quarter of chronic dialysis patients followed a decision to discontinue dialysis. Significant regional variation in discontinuation rates exists after adjusting for age, race, sex, and rural–urban status. Further research and analysis is needed on the cultural and economic factors that affect regional variation in health services utilization, especially in regard to the use of expensive medical services near the end of life.

## Background

Regional variation in cost and intensity of care has attracted the attention of researchers and health policy leaders [1-3]. As concerns about the cost of health care have grown, regional variation has been examined more closely. Overall, regional health care expenditures do not correlate well with quality of care. In 2003, Fisher et al. found that although Medicare enrollees in higher-spending regions received more care than those in lower-spending regions, they did not have better outcomes or satisfaction with care [4,5].

Regional variation in end-of-life care is of special interest, both because of the substantial Medicare expenditures in the last year of life [6-9], and because of concerns about overuse of services that may be both burdensome and offer little benefit to dying patients [10]. Significant regional variation has been documented in the use of feeding tubes [10,11], hospitalizations late in life [10], intensive care use in the last month of life [10], and other services and conditions [12] as well as in overall expenditures on end-of-life care [5,6]. The Dartmouth Atlas of Health Care documented substantial regional differences in the use of inpatient care, intensive care, physicians’ visits, and other services at the end of life, and concluded that *“…to the extent that end of life issues are addressed in practice, they are resolved in ways that depend on where the patient happens to live, not on the patient’s preferences or the power to extend life*” [13]. Many medical services – such as hospitalizations and consultations with specialists – are used more at the end of life in areas of the South and along the urban corridors on the East and West Coasts, when compared with use in Midwestern and Mountain regions [12].

The observed patterns of regional variation in health care utilization in the US may be affected by many factors, including the demographic and cultural characteristics of the regions’ populations; differences in the prevalence or severity of health problems, especially chronic diseases; and variations in practice patterns [4]. Greater utilization of medical care near the end of life has been associated with non-white race [6,8,10,14,15], younger age at the time of death [16-18], urban residency [19,20], and lack of advance directives [9]. Many of these factors also co-vary in populations. For example, black Americans make up a higher percentage of urban than rural populations, and complete advance directives at a lower rate than white Americans.

Several of the demographic characteristics associated with greater intensity of care at the end of life, such as non-white race and lower age at the time of death, are also more prevalent in the regions of the country known to have more intensive end-of-life care patterns. Therefore, adjustment for race, age, and rural–urban residence is necessary when assessing the magnitude of regional variation in medical care utilization at the end of life.

Data on end-stage renal disease (ESRD) and on discontinuation of dialysis prior to death may be used to examine regional variation in the end-of-life health care. In 1986, Neu and Kjellstrand reported that 22% of all ESRD deaths in a large regional dialysis program followed the discontinuation of dialysis therapy [21]. Over the ensuing 25 years, the discontinuation of dialysis prior to death has been examined extensively in both the US and Europe [22]. The proportion of ESRD deaths preceded by discontinuation of dialysis has been slowly rising [23]. Some regional variation in the discontinuation rates has been documented. For example, the percentage of ESRD deaths preceded by discontinuation of dialysis in the ESRD Network of New England was found to be 32-35% in 2001, when the national rate was 24% [24,25].

Regional variation in dialysis discontinuation prior to death may be affected by the same demographic factors that have been found to affect other end-of-life care patterns, especially age and race. The average age of dialysis patients varies significantly by region, with older populations of dialysis patients in areas such as New England, the Upper Midwest, and southern Florida than in the rest of the country [26]. The race of dialysis patients also varies significantly by region, with greater proportions of non-white patients in the South and along the East and West Coasts. The proportion of dialysis patients who reside in rural areas may also affect discontinuation rates, as rural populations have been found to use less intensive end-of-life care than urban counterparts [7,20]. Accordingly, regional dialysis discontinuation rates should be adjusted for age, race, and rural–urban residence.

In fact, the demographic factors associated with forgoing other medical interventions near the end of life are associated with dialysis discontinuation as well. Discontinuation rates are higher in whites than in other racial groups [27-29]. Discontinuation is also more common among women [23,29], among patients who are widowed or divorced [30], and those who are institutionalized [30,31]. Patients with depression [32], dementia [23,31,33] or malignancy [23] are more likely to discontinue dialysis, as are those with diabetes [29,34,35]. Higher pain and discomfort levels are also associated with higher discontinuation rates [30].

This study was undertaken to improve understanding of regional variation in health care intensity at the end of life, and to quantify regional variation in end-of-life care after adjusting for demographic differences between regions. Specifically, we examine regional variation in discontinuation of dialysis, after adjusting for race, age, sex and rural–urban residence.

## Methods

### Data sources and definitions

In this retrospective study we used data from the United States Renal Data System (USRDS), a national data registry for ESRD in the US. The USRDS incorporates longitudinal demographic and clinical data on all patients in the US Medicare ESRD program. An ESRD patient is a person with chronic renal failure who requires renal replacement treatment (dialysis or transplant) that has been certified by a physician at onset of ESRD, or when other evidence of chronic dialysis or a kidney transplant exists. Patients with acute renal failure who subsequently recover renal function are excluded from the USRDS [36].

Several USRDS standard analysis files were used in this study. The Patient File contains basic demographic information and ESRD-related data. The Treatment History File contains detailed treatment history including treatment periods, treatment modality and dialysis provider. The Residence File contains longitudinal records of ESRD patients’ residence and was used to determine ZIP codes of patients’ residence closest to the time of death. Records for individual patients were linked using USRDS identification numbers.

The ESRD Network Program, directed by the Centers for Medicare & Medicaid Services, consists of 18 ESRD networks, responsible for US states, territories and the District of Columbia. These networks service geographic areas based on the number and concentration of ESRD beneficiaries. We elected to use the 18 ESRD networks as the units for analysis of regional variation as the networks are used extensively in monitoring of the quality of chronic kidney disease care in the US. US territories were excluded from the analysis.

ZIP-code level files from the WWAMI Rural Health Research Center were used to define the Rural–urban Commuting Area (RUCA) codes [37] for each ESRD patient included in the study population. Based on standard US Census Bureau urbanized area and urban cluster definitions combined with commuter information, the RUCA codes classify all Census tracts by rural and urban status and relationships. Due to changes in the RUCA codes overtime, the level of rurality was estimated by linking ZIP codes from the Residence File to the version of ZIP code-level approximation RUCA codes closest to patient’s time of death: those deceased in 1995–1999 were assigned the version 1.11 RUCA codes based on 1990 Census commuting areas with 1998 ZIP codes [38]. Those deceased in 2000–2004 were assigned the version 2.0 RUCA codes based on 2000 Census commuting data and 2004 ZIP codes; and those deceased in 2005–2009 were assigned the version 2.0 RUCA codes with 2006 ZIP codes. The RUCA algorithm creates mutually exclusive categories that represent population density and affinity to nearby urban centers. We used 4-level aggregation of the RUCA codes: urban, large rural city/town, small rural town, or isolated small rural town to describe the level of rurality within the study population. The two categories “small rural town” and “isolated small rural town” were further collapsed into one category resulting in 3-level aggregation for the analysis.

### Study population

We limited the study population to patients enrolled in the Medicare ESRD program who resided in the 50 states of the US and the District of Columbia and who died between 1995 and 2009, inclusively. The date range was determined based on the availability of data on dialysis discontinuation prior to death. The death of an enrollee in the ESRD program is reported to the USRDS using Centers for Medicare & Medicaid Services form 2746. While the USRDS began using forms that provided an opportunity for recording whether or not dialysis had been discontinued prior to death in 1990, in the first years of the new reporting system (prior to 1995), discontinuation status was recorded on less than 20% of the forms. Between 1995 and 2000, more than 50% of forms had information regarding discontinuation status, and after 2000 nearly 90% of forms reported discontinuation status. In view of the uneven recording of discontinuation data from 1990 to 1994, these years were excluded from the analysis.

Additional criteria for inclusion were valid age and USRDS first service date, age at least 40 years old at the time of dialysis onset, length of dialysis prior to death at least 90 days, and known discontinuation of dialysis status. Patients with unknown age, sex, or race were excluded as were those for whom rural–urban status could not be determined. Patients who had a kidney transplant and who did not resume dialysis treatment prior to death were also excluded from the study population.

### Outcome variables and covariates

The outcome variable was discontinuation of dialysis prior to death. The primary independent variable for evaluation of regional variation in the rates of dialysis discontinuation prior to death was the ESRD network. Continuous covariates of interest were age at death, year of death, and years on dialysis. Categorical covariates included race (black, white, or other), sex (male or female), last treatment modality prior to death (hemodialysis or peritoneal dialysis), and RUCA-defined rurality (urban, large rural town, or small rural town/isolated small rural town).

### Statistical analysis

We used descriptive statistics to characterize the entire study population and stratified by the discontinuation of dialysis status, using means, standard deviations (SD) and medians for continuous variables and frequencies for categorical variables. We assessed bivariate relationships between patient characteristics and dialysis discontinuation status by using t-tests for means and Wilcoxon rank-sum tests for medians of continuous variables and chi-squared tests for categorical variables. The proportion of patients with discontinued dialysis by race and 5-year age group was calculated to ascertain the relationship between dialysis discontinuation, race and age.

We estimated the log-odds (logit) of discontinuation of dialysis with a logistic regression model with the main-effect explanatory variables given above. Empirical investigation of the raw data suggested a cubic effect for age at death on the log-odds of discontinuation of dialysis. We hypothesized that the effect of age at death may differ according to race and years on dialysis. For these reasons, the higher-order terms of quadratic and cubic age at death and three interaction terms of age at death by race, and years on dialysis were also included in the model. The ESRD network with the estimated probability of dialysis discontinuation closest to the median estimated probability for the overall study population was chosen as the reference in the estimation of odds ratios. Least-squares means (LS-means) of the log-odds for each network at the average value for each covariate in the model were also estimated. Following the inverse logit transformation, an estimate of the probability of discontinuation of dialysis as well as corresponding 95% confidence intervals, given the covariates in the model, was obtained for each network. Estimated probabilities of dialysis discontinuation for ESRD networks over the entire 15-year period from the model were grouped into quintiles and used for visual presentation of regional variation in the dialysis discontinuation.

The authors had full access to the data. All analyses were conducted by using SAS, version 9.2 or later (SAS Institute, Inc., Cary, North Carolina). The Essentia Health Institutional Review Board approved this study.

## Results

**Figure 1:**
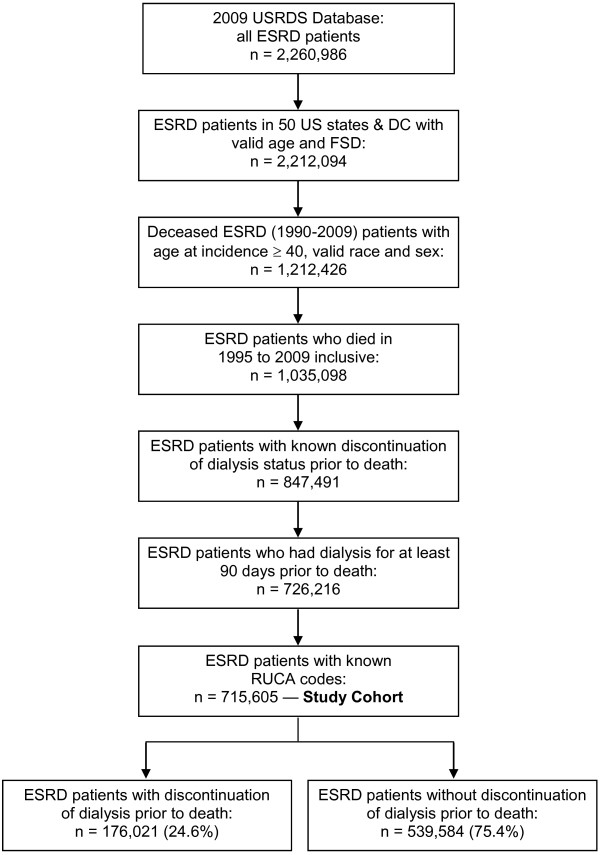
**Study cohort selection.** USRDS = United States Renal Data System; ESRD = end-stage renal disease; DC = the District of Columbia; FSD = USRDS first service date; RUCA codes = rural urban commuting area codes.

We identified 715,605 ESRD patients who died between 1995 and 2009 and had dialysis at least for 90 days prior to death, with known status of dialysis discontinuation. The selection of the study cohort is summarized in Figure 1. Table 1 reflects the demographic and dialysis-related characteristics of the study population by dialysis discontinuation status. Overall, the patients’ mean age at the time of death was 70.0 years (SD = 11.3); 47.7% were women; 65.5% were white, and 21.4% resided in rural areas. Hemodialysis was the last dialysis modality for the majority of patients (94.0%) and the median time on dialysis was 2.4 years (Table 1).

**Table 1:** Study population: characteristics of ESRD patients by dialysis discontinuation status prior to death (1995–2009)

**Variable**	**All patients**	**Patients with discontinuation of dialysis**	**Patients without discontinuation of dialysis**
No. of patients	715,605	176,021	539,584
Age at dialysis onset, mean (SD)	66.7 (11.92)	70.1 (11.20)	65.6 (11.94)
Age at death, mean (SD)	70.0 (11.34)	73.2 (10.65)	69.0 (11.37)
Age at death, n (%)			
40 – 59	140,670 (19.7)	21,133 (12.0)	119,537 (22.2)
60 – 69	179,489 (25.1)	36,937 (21.0)	142,552 (26.4)
70 – 79	235,667 (32.9)	63,532 (36.1)	172,135 (31.9)
80+	159,779 (22.3)	54,419 (30.9)	105,360 (19.5)
Male sex, n (%)	374,605 (52.3)	86,301 (49.0)	288,304 (53.4)
Race, n (%)			
White	468,979 (65.5)	138,203 (78.5)	330,776 (61.3)
Black	212,576 (29.7)	31,290 (17.8)	181,286 (33.6)
Asian	20,459 (2.9)	3,713 (2.1)	16,746 (3.1)
American Indian	9,479 (1.3)	2,168 (1.2)	7,311 (1.4)
Other	4,112 (0.6)	647 (0.4)	3,465 (0.6)
Location,* n (%)			
Urban	562,156 (78.6)	133,957 (76.1)	428,199 (79.4)
Large rural city/town	74,316 (10.4)	20,758 (11.8)	53,558 (9.9)
Small rural town	46,060 (6.4)	12,105 (6.9)	33,955 (6.3)
Isolated small rural town	33,073 (4.6)	9,201 (5.2)	23,872 (4.4)
Hemodialysis,** n (%)	672,623 (94.0)	166,308 (94.5)	506,315 (93.8)
Years on dialysis, mean (SD)	3.2 (2.90)	3.0 (2.77)	3.3 (2.94)
Years on dialysis, median (Q1, Q3)	2.4 (1.1, 4.5)	2.2 (0.9, 4.2)	2.5 (1.1, 4.6)

All of the differences between dialysis discontinuation groups are statistically significant at p < 0.001.

*ESRD patient residence based on RUCA codes (zip code approximation of Census tracks).

**Last treatment modality recorded prior to death.

ESRD = End Stage Renal Disease; SD=Standard Deviation; RUCA = Rural Urban Commuting Areas; Q1 and Q3 = first and third quartiles.

**Figure 2:**
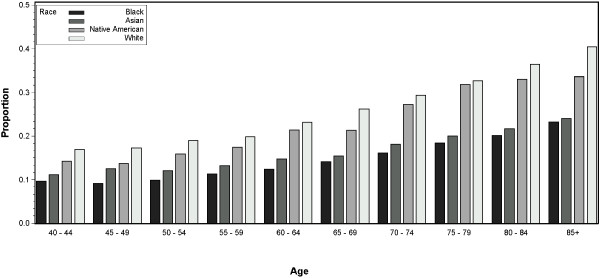
**Discontinuation of dialysis prior to death by race and age group.** Shown are unadjusted proportions of end-stage renal disease patients with discontinued dialysis (1995–2009).

Dialysis was discontinued prior to death in 176,021 (24.6 %) ESRD patients (Figure 1). Discontinuation was more frequent in those who were age 70 or older than in those under age 70 (29.83% vs. 18.14%, p < 0.001), and more frequent among women than men (26.3% vs. 23.0%, p < 0.001). Whites discontinued dialysis prior to death more frequently than blacks (29.5% vs. 14.7%, p < 0.001) or patients of other races (29.5% vs. 19.2%, p < 0.001). Residents of small and isolated rural towns discontinued dialysis at a slightly higher rate than residents of urban areas and large towns (26.9% vs. 24.3%, p < 0.001). The overall unadjusted rate of discontinuation of dialysis rose over the 15 years examined (p < 0.001). For example, the rate of dialysis discontinuation was 21.5% in 1995, 24.4% in 2002, and 27.8% in 2009. While the rates of dialysis discontinuation differed by race (p < 0.001) and age (p < 0.001), they consistently rose with age for all races (Figure 2). In each age group, blacks had lowest rates of dialysis discontinuation, and whites had the highest.

In each of the years examined, patients whose dialysis was discontinued prior to death were older on average – both at dialysis onset and at death – than those who died while still receiving dialysis. For example, the mean age at onset of dialysis among patients for whom dialysis was eventually discontinued prior to death was 64.5 years (SD = 10.6) in 1995, 69.9 years (SD = 11.1) in 2002, and 70.6 years (SD = 11.4) in 2009. However, over the examined 15 years, the mean age at onset of dialysis changed little for patients who were still on dialysis at the time of death: 64.8 years (SD = 11.3) in 1995, 65.7 years (SD = 11.9) in 2002, and 65.6 years (SD = 12.2) in 2009. The mean age at death increased steadily for patients for whom dialysis was discontinued: 71.3 years (SD = 10.1) in 1995, 72.9 years (SD = 10.6) in 2002, and 74.1 years (SD = 10.8) in 2009. Similar pattern in the mean age at death was observed for those for whom dialysis was not discontinued: 67.9 years (SD = 10.8) in 1995, 69.0 years (SD = 11.3) in 2002, and 69.3 years (SD = 11.6) in 2009. The time on dialysis had similar temporal trends. The mean length on dialysis for those with discontinuation of dialysis prior to death was 2.7 years in 1995, 2.9 years in 2002, and 3.3 years in 2009; while the mean length on dialysis for those who continued to receive dialysis was 3.0 years in 1995, 3.2 years in 2002, and 3.6 years in 2009.

**Figure 3:**
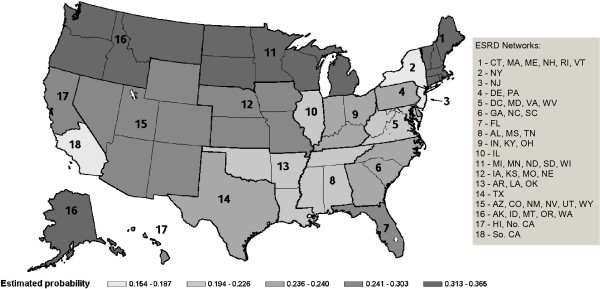
**Regional variation in discontinuation of dialysis in the US.** Shown are estimated probabilities of dialysis discontinuation in the US end-stage renal disease networks (1995–2009), adjusted for covariates and grouped into quintiles. Networks 2, 3 and 18 are in the lowest quintile, while networks 1, 11 and 16 populate the highest quintile.

The rates of dialysis discontinuation prior to death varied markedly by region of the country and are listed in Table 2. The observed (unadjusted) probability of dialysis discontinuation in the regions with highest rates (0.39 in network 16 – Alaska, Idaho, Montana, Oregon and Washington, and 0.36 in network 1 – New England) was twice as high as it was in the regions with the lowest rates (0.17 in network 18 – Southern California, and 0.18 in network 2 – New York). The magnitude of regional variation decreased somewhat after adjusting for the covariates in the multivariate model, but an approximate 2:1 ratio between the highest and lowest networks remained (0.37 in network 16 vs. 0.15 in network 18). The probabilities of dialysis discontinuation estimated from the logistic regression at average values of all covariates included in the model are shown in Table 2. The estimated probability for network 9 (Indiana, Kentucky and Ohio) – 0.236, was closest to the median estimated probability for the overall study population (0.233), and was used as a reference level for estimating odds ratios. The odds of discontinuation of dialysis prior to death for networks 6 (Georgia, North Carolina, South Carolina), 14 (Texas) and 17 (Hawaii and Northern California) did not statistically differ from the reference; the odds for the remaining networks were statistically significantly different relative to network 9. The probabilities of dialysis discontinuation, adjusted for covariates and grouped into quintiles over the entire 15 years of the study, are reflected in Figure 3.

**Table 2:** Regional variation in dialysis discontinuation prior to death in ESRD patients by ESRD network (1995–2009)

**ESRD network (US states included in the network)**	**Observed probability of discontinuation (95% CI)**	**Estimated probability**^**1 **^**of discontinuation (95% CI)**	**Adjusted odds ratio**^**2 **^**(95% CI)**
18 (Southern CA)	0.171 (0.167, 0.174)	0.154 (0.151, 0.158)	0.59 (0.57, 0.61)
2 (NY)	0.183 (0.180, 0.187)	0.170 (0.167, 0.174)	0.66 (0.64, 0.69)
3 (NJ)	0.193 (0.188, 0.198)	0.187 (0.182, 0.191)	0.74 (0.72, 0.77)
10 (IL)	0.202 (0.198, 0.206)	0.194 (0.190, 0.199)	0.78 (0.75, 0.81)
13 (AR, LA, OK)	0.205 (0.201, 0.209)	0.218 (0.214, 0.223)	0.90 (0.87, 0.93)
8 (AL, MS, TN)	0.199 (0.196, 0.203)	0.220 (0.216, 0.224)	0.91 (0.88, 0.94)
5 (DC, MD, VA, WV)	0.207 (0.204, 0.211)	0.226 (0.222, 0.230)	0.94 (0.91, 0.97)
6 (GA, NC, SC)	0.205 (0.202, 0.208)	0.236 (0.233, 0.240)	1.00 (0.97, 1.03)
9 (IN, KY, OH)	0.253 (0.250, 0.257)	0.236 (0.233, 0.240)	1.00*
4 (DE, PA)	0.269 (0.264, 0.274)	0.238 (0.234, 0.243)	1.01 (0.98, 1.04)
14 (TX)	0.250 (0.246, 0.254)	0.240 (0.236, 0.245)	1.02 (0.99, 1.05)
17 (HI, Northern CA)	0.248 (0.243, 0.253)	0.241 (0.236, 0.247)	1.03 (0.99, 1.06)
7 (FL)	0.257 (0.253, 0.261)	0.250 (0.246, 0.254)	1.08 (1.05, 1.11)
12 (IA, KS, MO, NE)	0.312 (0.307, 0.317)	0.288 (0.283, 0.294)	1.31 (1.27, 1.35)
15 (AZ, CO, NM, NV, UT, WY)	0.319 (0.314, 0.325)	0.303 (0.297, 0.308)	1.40 (1.36, 1.45)
1 (CT, MA, ME, NH, RI, VT)	0.361 (0.355, 0.368)	0.313 (0.307, 0.320)	1.48 (1.43, 1.53)
11 (MI, MN, ND, SD, WI)	0.332 (0.328, 0.336)	0.315 (0.311, 0.319)	1.48 (1.45, 1.52)
16 (AK, ID, MT, OR, WA)	0.395 (0.388, 0.401)	0.365 (0.358, 0.371)	1.85 (1.79, 1.92)

^1^Predicted probability of discontinuation of dialysis from LS-means estimates from the logistic regression (i.e. estimated at average values of all covariates included in the model);

^2^The odds of dialysis discontinuation for a ESRD network relative to reference network 9;

*Reference, network 9 with the rate of dialysis discontinuation closest to the median of estimated probability for the overall study population;

ESRD = End-Stage Renal Disease; CI = confidence interval.

## Discussion

The pattern of regional variation of dialysis discontinuation demonstrated in this study parallels the pattern of end-of-life care documented in the Dartmouth Atlas [13] and in other studies on variation in the intensity of care at the end-of-life [5,6,10]. The national maps from these studies present a consistent picture, with more intensive use of services in the South and in urban areas of the East and West Coasts, and less intensive use in the Upper Midwest, Mountain states, and New England. A study by Hirth et al. on geographic variation in Medicare ESRD expenditures [39] documented a similar national pattern, with the lowest ESRD expenditures in communities from the areas found to have high discontinuation rates in the current study. Similarly, O’Hare et al. found that ESRD patients living in areas of high end-of-life care intensity were less likely to have discontinued dialysis before death [40]. Overall, dialysis discontinuation rates provide a measure of intensity of end-of-life care that is consistent with measures used in prior work on geographic variation. The current study also demonstrates that significant regional variation persists after adjustment for demographic differences between regions.

The documentation of significantly lower rates of dialysis discontinuation in some regions of the country does not, in itself, mean that patient care in these regions is unnecessarily aggressive or intensive. However, the regional variation documented in the current study is not an isolated finding; the pattern of regional variation in intensity of end-of-life care is consistent. Whether the question is the use of feeding tubes in elderly patients with cognitive impairment [11], nursing home residents receiving potentially burdensome medical interventions near the end of life [10], or the overall level of intensity of medical care at the end of life [5], the same regions are consistently found to provide more aggressive (and expensive) end-of-life care. Future research directed at improving our understanding of the reasons why these regional variations exist is needed. Clearly, such variation cannot be explained by demographic differences between regions alone, as demonstrated by the current study.

We found that, overall, the average age of incident dialysis patients rose steadily over the 15 years of the study. However, there was little change in the age at dialysis onset among patients who remained on dialysis until death, while the age at dialysis onset of those whose dialysis was eventually discontinued rose significantly. This finding suggests that the use of dialysis in ever-older patients should be monitored closely, as co-morbidity and quality-of-life considerations are more prevalent in older patients, and contribute to many dialysis discontinuation decisions. The early start of dialysis in elderly patients, in particular, should be examined closely, as it is one of the principal factors contributing to the greying of the dialysis population, and does not consistently provide a mortality, morbidity or quality of life benefit [41-43].

In this study, we used the ESRD networks to examine regional variation in dialysis discontinuation rates. We recognized that the use of large geographic regions in the analysis obscured important small area variation within regions. However, we elected to use these large – mostly multi-state – regions with the goal of examining variability above the level of practice, institution, or community. The finding of significant regional variation after adjusting for age, race and rural–urban residence is interesting because other factors that have been found to be associated with discontinuation – female sex [23,29], malignancy [23], dementia [23], depression [32], and pain [44] – are not likely to vary substantially from region to region. The observed variation suggests that broader cultural variables – education, religion, values, and tradition – may have to be examined to arrive at a better understanding of regional variations in care. In efforts to rein in health care costs, policy makers will need to consider both small area variation – often reflecting local, institutional or practice patterns that lead to variation – and regional variation as documented in the current study. Future research should be directed not only at how small area differences in medical practice affect end-of-life care, but on how regional traditions and culture affect patterns and preferences for care.

Further study of regional variation in end-of-life practices may provide insights into how end-of-life care may be improved in general. Communities where aggressive medical interventions are used sparingly near the end of life may constitute “laboratories” for examining how medical and quality-of-life considerations may be balanced to achieve better end-of-life experiences. Conversely, examination of communities where more aggressive end-of-life care is routine may lead to a better understanding of the patient, provider, and cultural factors that are associated with such intensive – some might say nonbeneficial – practices. Other factors that may affect end-of-life care practices, such as provider-induced demand for services, the availability and utilization of hospice services, personal values, and religious beliefs are challenging to study, but may provide valuable insights into regional variation.

The strengths of the current study include the examination of a nearly “100% sample” from the USRDS’s national data spanning 15 years, and the examination of regional variation after adjustment for key demographic variables. The study also has several potential limitations. First, the data on discontinuation of dialysis in this study came from the death notification form utilized by the USRDS. This form does not provide clinicians with opportunities to clarify whether dialysis was discontinued with the intention of allowing natural death to occur, or because death from another cause was imminent. The indication that dialysis therapy was discontinued prior to death does not signify that the patient died from the discontinuation of dialysis. Second, although the ESRD data reported to the USRDS is thought to be complete and current, it was not be possible to determine how many cases of ESRD deaths occurred but went unreported, and – more significantly – how many ESRD deaths were reported but without a record of a decision to terminate dialysis prior to death. Third, for the entire 15 years examined in the study, more than 82% of forms had information regarding discontinuation status. The proportion of death notification forms with known dialysis discontinuation status was homogeneous for all networks during 2000 – 2009, but greater variation was observed before 2000, suggesting the potential for bias due to differences either in the characteristics of patients for whom dialysis discontinuation status data was not available, or in networks with differences in reporting. A sensitivity analyses conducted for a 10-year cohort (2000–2009) compared to the overall 15-year cohort (1995–2009) showed no appreciable differences between the cohorts beyond temporal influences consistent with the overall findings of the study. That is, patients in the 15-year cohort tended to be slightly younger at the time of death, with slightly earlier withdrawal than those in the 10-year cohort. The 15-year cohort, which included 5 years of earlier data, was slightly less urbanized than the 10-year cohort, reflecting subtle demographic shifts that occurred during the observation window. The results of the multivariate analysis on the 10-year cohort were also consistent with the outcomes of the results obtained for the full 15-year cohort, supporting our conclusion that there is no discernible bias attributable to the differential in the reporting rates over time. Finally, this study used ESRD Networks as geographic units of analysis. Accordingly, the effects of provider, facility and community variation and the effects of clustering of patients within regions were not addressed. The effects of such variables are best assessed using the methods that have been established for examining small area variation; this was beyond the scope of the current study.

## Conclusions

Discontinuation of dialysis prior to death was common in the US between 1995 and 2009. The deaths of nearly one quarter of chronic dialysis patients followed a decision to discontinue dialysis. Significant regional variation in discontinuation rates exists after adjusting for age, race, sex, and rural–urban status. Further research and analysis is needed on the cultural and economic factors that affect regional variation in health services utilization, especially in regard to the use of expensive medical services near the end of life.

## Competing interests

The authors declare that they have no conflicts of interests.

## Authors’ contributions

CEG conceived of the study idea, participated in the interpretation of results, drafted parts of the manuscript, and provided critical revisions to the current manuscript. IVH participated in the development of the study, study analysis and data interpretation, drafted parts of the manuscript, and provided critical revisions to the current manuscript. BPJ conducted all analyses, drafted parts of the manuscript, participated in interpretation of the results, and provided critical revisions to the current manuscript. All authors read and approved the final manuscript.

## Pre-publication history

The pre-publication history for this paper can be accessed here:

http://www.biomedcentral.com/1471-2318/13/39/prepub
